# Excessive dietary histamine inhibits growth performance and impairs intestinal and hepatic health in juvenile American eel (*Anguilla rostrata*)

**DOI:** 10.3389/fphys.2026.1797772

**Published:** 2026-04-10

**Authors:** Yichuang Xu, Qiya Xu, Xinyu Hu, Jinyue Yang, Shaowei Zhai

**Affiliations:** 1 Fisheries College, Jimei University, Xiamen, China; 2 Engineering Research Center of the Modern Technology for Eel Industry, Ministry of Education, Xiamen, China; 3 Department of Modern Agriculture, Linyi Vocational University of Science and Technology, Linyi, China; 4 College of Ocean Food and Biological Engineering, Jimei University, Xiamen, China

**Keywords:** American eels, growth, histamine, intestine, liver

## Abstract

**Introduction:**

Histamine commonly accumulates in animal–derived feed ingredients and is detrimental to aquaculture species, but the adverse consequences of dietary histamine remain inadequately characterized. Here, we explored the physiological influences of different dietary histamine levels on the growth performance, as well as the intestinal and hepatic health, of American eels, a fish species intolerant to histamine.

**Methods:**

Five diets were prepared with total histamine levels of 48.94, 198.19, 355.31, 492.44, and 641.37 mg kg^-1^, respectively. Following a 10-week feeding trial, growth performance, intestinal and hepatic morphology, antioxidant capacity, intestinal microbiota composition, and hepatic metabolites were analyzed.

**Results:**

American eels receiving diets with histamine levels exceeding 355.31 mg kg⁻¹ exhibited reduced growth performance, feed utilization, immune competence, intestinal mucosal and hepatocellular integrity, intestinal and hepatic antioxidant capacity, and intestinal digestive enzymes activities, along with elevated intestinal and hepatic malondialdehyde contents, compared to those fed the diet containing 48.94 mg kg^-1^ histamine. Omics profiling further indicated that, relative to the dietary histamine level of 48.94 mg kg^-1^, the 641.37 mg kg^-1^ dietary histamine restructured intestinal microbiota composition by reducing the abundance *Enterococcus* and *Rhodococcus* while increasing the levels of *Haliangium*, *Stenotrophomonas*, and *Blastomonas*; and affected hepatic aminoacyl–tRNA biosynthesis, glycine/serine/threonine metabolism, nicotinate/nicotinamide metabolism, and valine/leucine/isoleucine biosynthesis in American eels. Broken–line regression analysis of weight gain rate identified 260.11 mg kg^-1^ as the maximum tolerable dietary histamine level for American eels.

**Conclusion:**

These findings provide crucial insights regarding dietary histamine levels and underscore the importance of restricting histamine content in American eel diets.

## 1 Introduction

Food safety is a critical concern not only for human health, but also for the wellbeing of farmed animals and economic sustainability of farming industry (Zhang et al., 2021; Zhao et al., 2022; Li et al., 2023). While aquaculture has been the most rapidly expanding sector in global farming industry over the past two decades, it simultaneously faces acute challenges regarding feed safety. Anti–nutritional factors, contaminants, and other toxicants in feed is detrimental to the health and growth of aquatic animals, and ultimately compromising their quality and economic value (Zhai et al., 2020; Zhang et al., 2021; Zhao et al., 2022; Li et al., 2023; Ding et al., 2025). Therefore, analyzing feed contaminant toxicity and assessing their tolerance limits in aquatic animals can optimize feed formulations and promote sustainable aquaculture development.

Among these feed hazards, the quantity and harm of histamine have attracted increasing attention (Li et al., 2018; Zhai et al., 2020). Histamine, a heterocyclic amine, is generated by the decarboxylation of L–histidine catalyzed by histidine decarboxylase and represents one of the most significant toxicants in protein–rich animal products (De Palma et al., 2022). Histamine accumulation correlates with the progress of meat decomposition and is particularly elevated in high–protein ingredients that stored under improper conditions or contaminated with microorganisms (Ashiq et al., 2025). Consequently, histamine usually serves as a crucial biomarker for feed quality and safety, directly reflecting the freshness and hygiene of the diet (Ashiq et al., 2025). Nevertheless, the pathophysiological consequences of dietary histamine remain inadequately characterized, representing a critical knowledge gap for feed safety assurance.

Dietary histamine is absorbed across the intestinal epithelium, and the excess histamine enters the systemic circulation (De Palma et al., 2022; Schnedl and Enko, 2021). The intestine is generally recognized as a primary target of histamine intoxication (Li et al., 2021; Liu et al., 2021a; Zhai et al., 2020). Previous studies demonstrated that excessive dietary histamine causes structural injury, oxidative damage, and microbiota dysbiosis of intestine in fish (Li et al., 2021; Liu et al., 2021a; Zhai et al., 2020). Recent findings further indicated that the effects of histamine extend beyond the gastrointestinal tract to affect systemic physiological functions (Schnedl and Enko, 2021). It has been found that excessive dietary histamine leads to hepatic structural damage, muscle sclerosis, immunosuppression, reduced feed efficiency, and inhibited growth performance of fish (Liu et al., 2022; Zhai et al., 2020; Zhang et al., 2023). Moreover, the tolerance thresholds of dietary histamine vary significantly among fish species. Liu et al. (2022) reported that dietary histamine exceeding 248.87 mg kg^-1^ inhibited the growth and compromised intestinal structural integrity of striped catfish (*Pangasianodon hypophthalmus*). By comparison, growth inhibition in grouper (*Epinephelus coioides*) was observed at significantly higher dietary histamine levels of 2500 mg kg^-1^ (Liu et al., 2021b). Therefore, identifying the specific adverse effects and precision limitation of dietary histamine levels is of crucial significance for supporting the growth and health of fish.

Eels are globally significant economic aquaculture species with considerable nutritional value. China is the primary country for eel aquaculture globally, and the American eel stands as the dominant farmed species nationally. Traditionally, white fishmeal is the main dietary protein source for American eels, but rising costs and supply shortages of white fishmeal have led to the increased inclusion of brown fishmeal in their feed (Zhai et al., 2020; Lu et al., 2023; Cai et al., 2025). The high histamine content resulting from elevated proportions of dietary brown fishmeal has been shown to compromise the growth of American eels (Zhai et al., 2020; Lu et al., 2023; Cai et al., 2025). Nevertheless, the toxicological influences of dietary histamine and the tolerable dietary histamine thresholds for eels remain poorly elucidated, hindering feed optimization and the sustainable aquaculture of them. Accordingly, this study aimed to determine the precise tolerance threshold in American eels by evaluating growth performance and physiological health. The findings are expected to provide evidence–based recommendations for histamine or brown fishmeal restriction levels in diets for American eels and to promote aquaculture development for this species, with broader implications for related species.

## 2 Materials and methods

### 2.1 Experimental design and diet formulation

**Table 1** details the ingredients and proximate composition of the basal formulation, which utilized white fishmeal as the primary protein source and microencapsulated fish oil powder as the principal lipid component. Five isonitrogenous and isolipidic diets were formulated by supplementing histamine (#S20188, Shanghai Yuanye Bio–Technology Co., Ltd, Shanghai, China) at nominal levels of 0, 150, 300, 450, and 600 mg kg^-1^, respectively. The analyzed total histamine concentrations were 48.94, 198.19, 355.31, 492.44, and 641.37 mg kg^-1^, respectively. Among them, the diet containing 48.94 mg kg^-1^ histamine was designated as the control diet. The experimental diet preparation followed the protocol of Xu et al. (2025). Briefly, all ingredients were ground, sieved, blended with the required histamine dose, homogenized, and then stored at -20 °C until use. Immediately before feeding, the powdered diet was mixed with distilled water to obtain a uniform dough–like consistency.

**Table 1:** Formulation and proximate composition (% of dry weight basis) of the basic diet.

Ingredients (%)		Proximate analysis %, dry weight	
White fishmeal	66.00	Crude protein	46.48
α-starch	23.50	Crude lipid	5.29
Microencapsulated fish oil powder	5.00	Ash	11.46
Extruded soybean	2.00	Calcium	3.10
Yeast powder	2.00	Phosphorus	2.04
Ca(H_2_PO_4_)_2_	0.50		
Choline chloride	0.50		
Vitamin premix^a^	0.40		
Mineral premix^b^	0.10		

a Vitamin premix (IU or mg kg^-1^ diet): Vitamin A (VA) 14000.00 IU, Vitamin C (VC) 200.00 mg, Vitamin D (VD) 3200.00 IU, Vitamin E (VE) 40.00 mg, Vitamin K (VK) 16.00 mg, Vitamin B1 (VB_1_) 20.00 mg, Vitamin B6 (VB_6_) 16.00 mg, folic acid 4.80 mg, nicotinic acid 80.00 mg.

b Minerals premix (mg kg^-1^diet): CuCl_2_ 12.05, FeCl_2_·H_2_O 666.67, MnSO_4_·H_2_O 94.34, ZnSO_4_·H_2_O 202.89, Ca(IO_3_)_2_ 2.59, Na_2_SeO_3_ 0.89, CoSO_4_ 3.64.

### 2.2 Feeding and sampling

After a 4–week acclimation period, 450 healthy and uniform–sized American eels (11.78 ± 0.30 g per fish, mean ± S.D.) were randomly distributed among 15 tanks (30 fish per tank, triplicate tanks per treatment). The experiment utilized a recirculating aquaculture system (RAS). American eels were fed daily at approximately 2% body weight., and residual feed was removed. Approximately one–third of the water volume was renewed each day. Temperature, pH, dissolved oxygen, ammonia nitrogen, and nitrite were maintained at 24–27 °C, 7.00–7.60, 7.10–9.20 mg L^-1^, 0.30–0.70 mg L^-1^, and 0.02–0.06 mg L^-1^, respectively.

At the end of the 10–week feeding period, American eels were fasted for 24 h and then anesthetized using a eugenol–ethanol solution (1:9, v/v; 50 mL L^-1^) before weighing and counting. Blood was drawn from the caudal vasculature, held at 4 °C for 24 h, and centrifuged (4 °C, 3000 rpm, 10 min) to isolate serum, which was stored at –80 °C for further testing. For each tank, intestinal tissue from three fish was sampled for microbial analyses; hepatic samples from three fish were taken for metabolomic analysis; intestinal and hepatic samples from ten fish were obtained to determine enzyme activities and antioxidant–related indicators, tissues from ten fish within each tank were pooled and homogenized to obtain a single composite sample per tank; intestinal and hepatic samples from one eel were preserved for histological examination.

### 2.3 Analytical methods

#### 2.3.1 Growth performance, feed utilization, and morphometric indices

The final total weight of American eels from each tank was determined. Ten fish were randomly selected from each tank to measure morphometric indices, including the hepatosomatic index (HSI), viscerosomatic index (VSI), and condition factor (CF). The weight gain rate (WGR), specific growth rate (SGR), feed intake (FI), feed efficiency (FE), survival rate (SR), HSI, VSI, and CF were calculated faccording to our previous studies (Lu et al., 2023; Xu et al., 2023), as follows:

IBW (g fish^-1^) = initial body weight;
FBW (g fish^-1^) = final body weight;
WGR (%) = (FBW - IBW)/IBW × 100;
SGR (% d^-1^) = (Ln FBW - Ln IBW)/days × 100;
FI (g fish^-1^) = the sum of feed consumed by fish throughout the experiment/fish number per tank;
FE (%) = (FBW - IBW) (g)/FI (g) × 100;
SR (%) = final fish number/initial fish number × 100;
HIS (%) = liver weight (g)/body weight (g) × 100;
VSI (%) = viscera weight (g)/body weight (g) × 100;
CF (g cm^-3^) = body weight (g)/body length^3^ (cm^3^) × 100.

#### 2.3.2 Proximate composition

Proximate composition of the basal diets was determined following AOAC (2011). Briefly, moisture was measured after 48h of freeze–drying (Labogene, Denmark), crude protein and crude lipid levels were quantified via the Kjeldahl method and Soxhlet extraction method, respectively, and ash content was determined by combustion at 550 ± 20 °C. Calcium and phosphorus levels were analyzed using inductively coupled plasma atomic emission spectrometry.

#### 2.3.3 Analysis of serum biochemical parameters

The activities of acid phosphatase (ACP, #A060–1–1), alkaline phosphatase (AKP, #A059–2–2), diamine oxidase (DAO, #A088–2–1), glutamic–oxalacetic transaminase (GOT, #C010–1–1) and glutamic–pyruvic transaminase (GPT, #C009–1–1) were determined using kinetic colorimetric methods, while the concentration of D–lactic acid (D–lac, #A019–3–1) was measured using an endpoint colorimetric assay. All reactions were monitored using a spectrophotometer (Infinite 200 PRO, Tecan Group Ltd., Männedorf, Switzerland). Commercial assay kits were purchased from Nanjing Jiancheng Bioengineering Institute (Nanjing, China). These kits are based on standardized enzymatic or colorimetric biochemical reactions and are supplied with manufacturer-provided validation parameters. In the present study, assay performance was verified through routine laboratory quality control procedures, and intra-assay coefficients of variation (CV) were maintained below 5%.

#### 2.3.4 Serum lipid profile

The levels of high–density lipoprotein cholesterol (HDL–C, #A112–1–1), low–density lipoprotein cholesterol (LDL–C, #A113–1–1), total cholesterol (TC, #A111–1–1), and triglyceride (TG, #A110–1–1) were measured using endpoint colorimetric kits (Nanjing Jiancheng Bioengineering Co., Ltd., Nanjing, China) with spectrophotometric detection (Infinite 200 PRO, Tecan), following the manufacturer’s instructions. Assay performance specifications are available in the manufacturer’s documentation for each catalog number, and within-run repeatability was verified in-house, with intra-assay CV maintained below 5%.

#### 2.3.5 Serum immunological analysis

The levels of complement 3 (C3, #H186–1–2) and immunoglobulin M (IgM, #H109–1–2) were quantified using immunoturbidimetric assays. These assays were measured using commercial assay kits (Nanjing Jiancheng Bioengineering Co., Ltd.). All procedures were performed strictly according to the manufacturer’s instructions. Routine intra-assay quality control was conducted prior to sample evaluation, with intra-assay CV maintained below 5%.

#### 2.3.6 Histological and ultrastructural examination

Histological specimens of the intestine and liver were processed as described by Xu et al. (2023). Sections were stained with hematoxylin–eosin (H&E) and visualized under a light microscope (BX80–JPA, Olympus, Tokyo, Japan). Morphometric measurements were subsequently conducted using Image–Pro Plus 6.0 software (Media Cybernetics, Silver Spring, MD, USA). The villus height was measured from the tip of the villus to the villus–crypt junction. For each image, 3 well–oriented and intact villi were randomly selected and measured.

Scanning electron microscopy (SEM) analysis was used to visualize the ultrastructure of intestinal microvilli. Intestinal segments were fixed in 2.5% (v/v) glutaraldehyde prepared in 0.1 M phosphate buffer at room temperature for 2 h. Following primary fixation, intestinal segments were rinsed three times with 0.1 M phosphate buffer and post-fixed in 1% osmium tetroxide at room temperature for about 2 h in the dark. Samples were then washed three times with phosphate buffer and dehydrated through a graded ethanol series followed by two changes of 100% ethanol. Subsequently, samples were immersed in isoamyl acetate for 15 min and dried using a critical point dryer. The dried specimens were attached to conductive carbon tape and sputter-coated with gold. Finally, the morphological features of the intestinal mucosa were observed using a scanning electron microscope (#SU8100, Hitachi High-Tech Corporation, Tokyo, Japan).

#### 2.3.7 Analysis of intestinal digestive enzymes

Approximately 0.5 g of intestinal tissue were collected from each tank and rinsed with ice-cold physiological saline to remove residual blood. The tissues were then placed in centrifuge tubes and homogenized in pre-cooled physiological saline at a ratio of 1:9 (w/v) using a tissue grinder (Tissuelyser-24, Shanghai Jingxin Industrial Development Co., Ltd., Shanghai, China). The homogenates were centrifuged at 3000 rpm for 10 min at 4 °C, and the resulting supernatants were collected for subsequent analysis. The activities of digestive enzymes, including amylase (#C016–1–1), lipase (#A054–2–1), and protease (#A080–2–2), were quantified using commercial endpoint colorimetric assay kits (Nanjing Jiancheng Bioengineering Co., Ltd.) via a spectrophotometer (Infinite 200 PRO, Tecan). All procedures were performed strictly according to the manufacturer’s instructions. Routine quality control was conducted prior to sample evaluation, with intra-assay CV maintained below 5%. Tissue protein concentrations were determined using the Coomassie brilliant blue method, and enzyme activities were subsequently normalized to these protein levels.

#### 2.3.8 Assessment of antioxidant capacity

The preparation of tissue homogenates followed the protocol described in Section 2.3.7. The activities of superoxide dismutase (SOD, #A001–1–1), catalase (CAT, #A007–1–1), and glutathione peroxidase (GSH–Px, #A005–1–2), alongside total antioxidant capacity (T–AOC, #A015–2–1) and the levels of malondialdehyde (MDA, #A003–1–2), vitamin C (VC, #A009–1–1), and vitamin E (VE, #A008–1–1), were quantified using commercial endpoint colorimetric assay kits (Nanjing Jiancheng Bioengineering Co., Ltd.) via a spectrophotometer (Infinite 200 PRO, Tecan). All procedures were performed strictly according to the manufacturer’s instructions. Routine quality control was conducted prior to sample evaluation, with intra-assay CV maintained below 5%. Tissue protein concentrations were determined using the Coomassie brilliant blue method, and all enzyme activities and biomarker levels were subsequently normalized to the protein content of each sample.

#### 2.3.9 Analysis of microbial profiling

Approximately 50 mg of intestinal tissue was transferred to a centrifuge tube, minced, and homogenized. The homogenate was then centrifuged at 1000 rpm for 5 min at 4 °C. Microbial DNA was isolated from intestinal contents using a commercial kit (Omega Bio–Tek, Norcross, GA, USA). DNA integrity was assessed by 1% agarose gel electrophoresis, and concentration and purity were quantified by spectrophotometry. The V3–V4 region of the bacterial 16S rRNA gene was amplified using the primers 338F (5’-ACTCCTACGGGAGGCAGCAG-3’) and 806R (5’-GGACTACNNGGGTATCTAAT-3’). PCR was performed and amplified products underwent sequencing on the Illumina MiSeq PE300 platform. Raw sequencing data were deposited in the NCBI SRA database (ID: PRJNA1415989). Sequence processing was conducted using QIIME (v1.8.0) to demultiplex, assemble paired–end reads, and quality–filter sequences. Operational taxonomic units (OTUs) were clustered at 97% similarity and taxonomically classified against the Silva database. Alpha diversity indices were calculated using Mothur (v1.31.2).

#### 2.3.10 Metabolomics analysis

The liver tissue samples were weighed and transferred into centrifuge tubes. A tissue extraction solution, prepared with 75% methanol and 25% chloroform (v/v, 9:1 ratio), was precisely added. After homogenization using a tissue grinder, extracts were centrifuged (12, 000 rpm, 20 min, 4 °C). Following centrifugation, the supernatant was collected and underwent concentration. The concentrated solution was filtered, and the filtrate was transferred to an autosampler vial. Metabolomics analysis was performed using a Thermo Vanquish liquid chromatography (LC) system. The mobile phase comprised 0.1% formic acid and 0.1% ethyl isocyanide formate in positive ion mode, and 5 mM ammonium formate with acetonitrile in negative ion mode. To monitor analytical system stability, quality control (QC) samples were interspersed at predetermined intervals throughout the sample sequence. Detection was performed by electrospray ionization (ESI) in both positive and negative modes.

### 2.4 Statistical analysis

Data analyses were performed with SPSS 26.0 (IBM, Armonk, NY, USA). Quantitative data are reported as means ± standard deviation (S.D.). The normality of the data was assessed using the Shapiro-Wilk test. Differences among treatment groups were evaluated by a one–way ANOVA, followed by Duncan’s multiple range test for post–hoc comparisons. For comparisons between two groups, independent Student’s *t*–test was employed. Statistical significance was established at *P* < 0.05. Prior to analysis, percentage data falling outside the 30–70% range underwent arcsine square root transformation to normalize distribution using the formula: Y = arcsin(√p), where p represents the proportion expressed as a decimal. Orthogonal polynomial contrasts were performed to evaluate linear and quadratic responses of primary variables to graded dietary histamine levels. The dietary histamine tolerance threshold was estimated using broken-line regression analysis.

## 3 Results

### 3.1 Growth performance, feed utilization, and morphometrics

**Figure 1:**
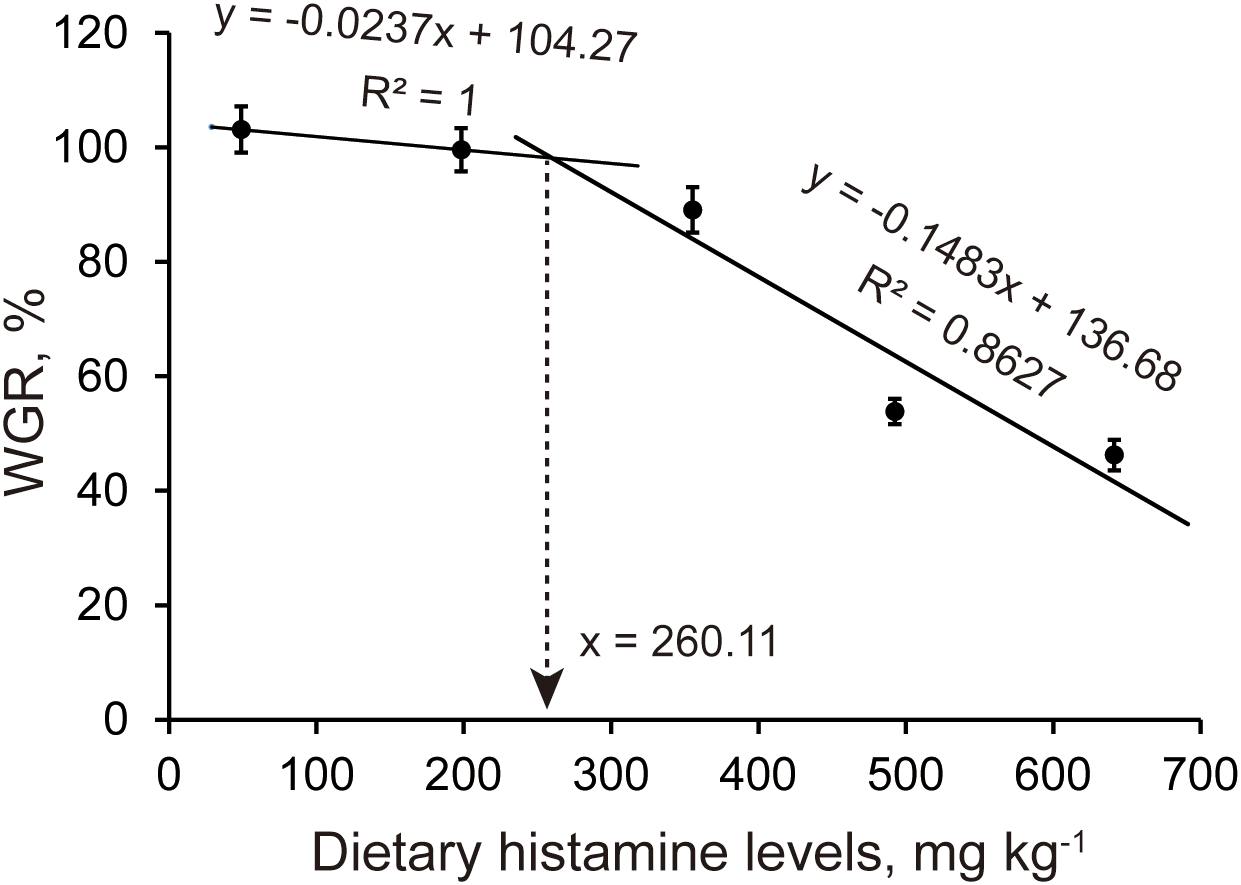
The relationship between dietary histamine levels and the WGR of juvenile American eels. Values are shown as mean ± S.D. (n = 3).

Initially, we examined the impacts of graded dietary histamine levels on the growth performance, feed utilization, and morphometric indices of American eels (**Tables 2**, **3**). Orthogonal contrast analysis revealed that FBW (*P_linear_*< 0.001, *P_quadratic_*= 0.001), WGR (*P_linear_*< 0.001, *P_quadratic_*= 0.001), SGR (*P_linear_*< 0.001, *P_quadratic_*< 0.001), FI (*P_linear_*< 0.001, *P_quadratic_*= 0.007), and FE (*P_linear_*< 0.001, *P_quadratic_*= 0.002) decreased both linearly and quadratically with increasing dietary histamine levels. Meanwhile, CF (*P_linear_*= 0.003, *P_quadratic_*= 0.376) showed a significant linear response to increasing dietary histamine levels. Relative to the control diet, dietary histamine levels exceeding 355.31 mg kg^-1^ significantly lower FBW, WGR, SGR, FE, and CF. No significant differences were observed in these parameters between American eels fed diets containing either 48.94 or 198.19 mg kg^-1^ histamine. American eels fed diets containing either 492.44 or 641.37 mg kg^-1^ histamine showed lower FI in comparison to those receiving diets with less than 355.31 mg kg^-1^ histamine. Neither the HSI nor the VSI was significantly influenced by dietary histamine treatment. Broken–line regression analysis of WGR established a maximum tolerable dietary histamine threshold of 260.11 mg kg^-1^ for American eels (**Figure 1**). Thus, excessive dietary histamine levels inhibit growth performance and feed utilization, as well as reduced condition factor, in American eels.

**Table 2:** Effects of dietary histamine on the growth performance, feed utilization, and somatic indices of juvenile American eel.

Item	Dietary histamine levels (mg kg^-1^)				
	48.94	198.19	355.31	492.44	641.37
IBW (g fish^-1^)	11.80 ± 0.03	11.78 ± 0.02	11.81 ± 0.01	11.81 ± 0.03	11.80 ± 0.02
FBW (g fish^-1^)	23.97 ± 0.48^d^	23.55 ± 0.45^d^	22.31 ± 0.47^c^	18.16 ± 0.26^b^	17.26 ± 0.32^a^
WGR (%)	103.11 ± 4.03^d^	99.58 ± 3.77^d^	89.07 ± 4.00^c^	53.87 ± 2.20^b^	46.24 ± 2.67^a^
SGR (% d^-1^)	1.01 ± 0.03^d^	0.99 ± 0.03^d^	0.91 ± 0.03^c^	0.62 ± 0.02^b^	0.54 ± 0.03^a^
FI (g fish^-1^)	12.40 ± 0.21^c^	12.67 ± 0.11^c^	11.92 ± 0.45^c^	11.07 ± 0.57^b^	9.87 ± 0.48^a^
FE (%)	95.15 ± 1.67^c^	92.80 ± 3.89^bc^	88.23 ± 3.85^b^	57.53 ± 3.59^a^	55.25 ± 3.46^a^
SR (%)	97.78 ± 1.92	95.56 ± 1.92	95.56 ± 5.09	93.33 ± 6.67	93.33 ± 8.82
HSI (%)	1.25 ± 0.16	1.28 ± 0.06	1.30 ± 0.08	1.27 ± 0.16	1.21 ± 0.21
VSI (%)	4.18 ± 0.40	4.59 ± 0.10	4.36 ± 0.31	4.40 ± 0.33	4.47 ± 0.47
CF (g cm^-3^)	0.17 ± 0.0^b^	0.16 ± 0.01^ab^	0.16 ± 0.02^a^	0.15 ± 0.01^a^	0.14 ± 0.01^a^

All values are shown as mean ± S.D. (n = 3). Letters (a-d) denote significance at P < 0.05.

**Table 3:** Polynomial contrasts reveal linear and quadratic dose-response patterns of growth performance to dietary histamine.

Item	*P_linear_*	*P_quadratic_*
FBW (g fish^-1^)	<0.001	0.001
WGR (%)	<0.001	0.001
SGR (% d^-1^)	<0.001	<0.001
FI (g fish^-1^)	<0.001	0.007
FE (%)	<0.001	0.002
CF (g cm^-3^)	0.003	0.376

*P_linear_*, linear contrast *P* values. *P_quadratic_*, quadratic contrast *P* values.

### 3.2 Immune function

**Figure 2:**
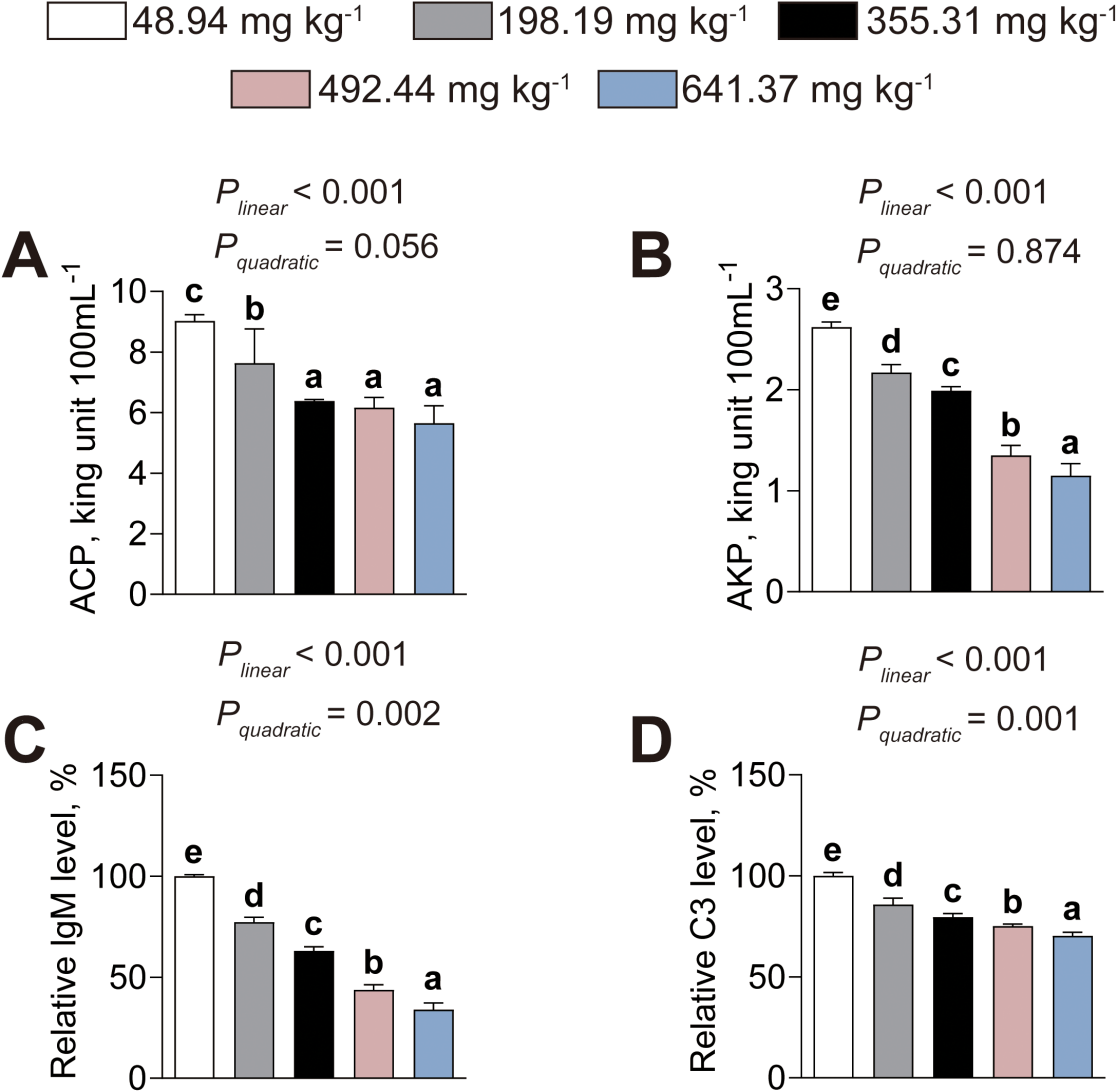
Effects of dietary histamine levels on the immune function of juvenile American eels. **(A)** Serum ACP activity. **(B)** Serum AKP activity. **(C)** Serum lgM concentration. **(D)** Serum C3 content. Values are shown as mean ± S.D. (n = 3). Letters (a–d) denote significance at *P* < 0.05.

**Figure 2** shows the immunomodulatory effects of dietary histamine levels on American eels. The activities of serum ACP (*P_linear_*< 0.001, *P_quadratic_*= 0.056) and AKP (*P_linear_*< 0.001, *P_quadratic_*= 0.874) exhibited significant linear decreases with increasing dietary histamine (**Figures 2A, B**). Concurrently, the levels of serum IgM (*P_linear_*< 0.001, *P_quadratic_*= 0.002) and C3 (*P_linear_*< 0.001, *P_quadratic_*= 0.001) decreased both linearly and quadratically with increasing dietary histamine (**Figures 2C, D**). Dietary histamine levels exceeding 198.19 mg kg^-1^ significantly lower serum ACP and AKP activities, as well as serum lgM and C3 levels, compared to those receiving the control diet (**Figures 2A–D**). Therefore, excessive dietary histamine levels compromise immune function of American eels.

### 3.3 Serum lipid indices

**Figure 3:**
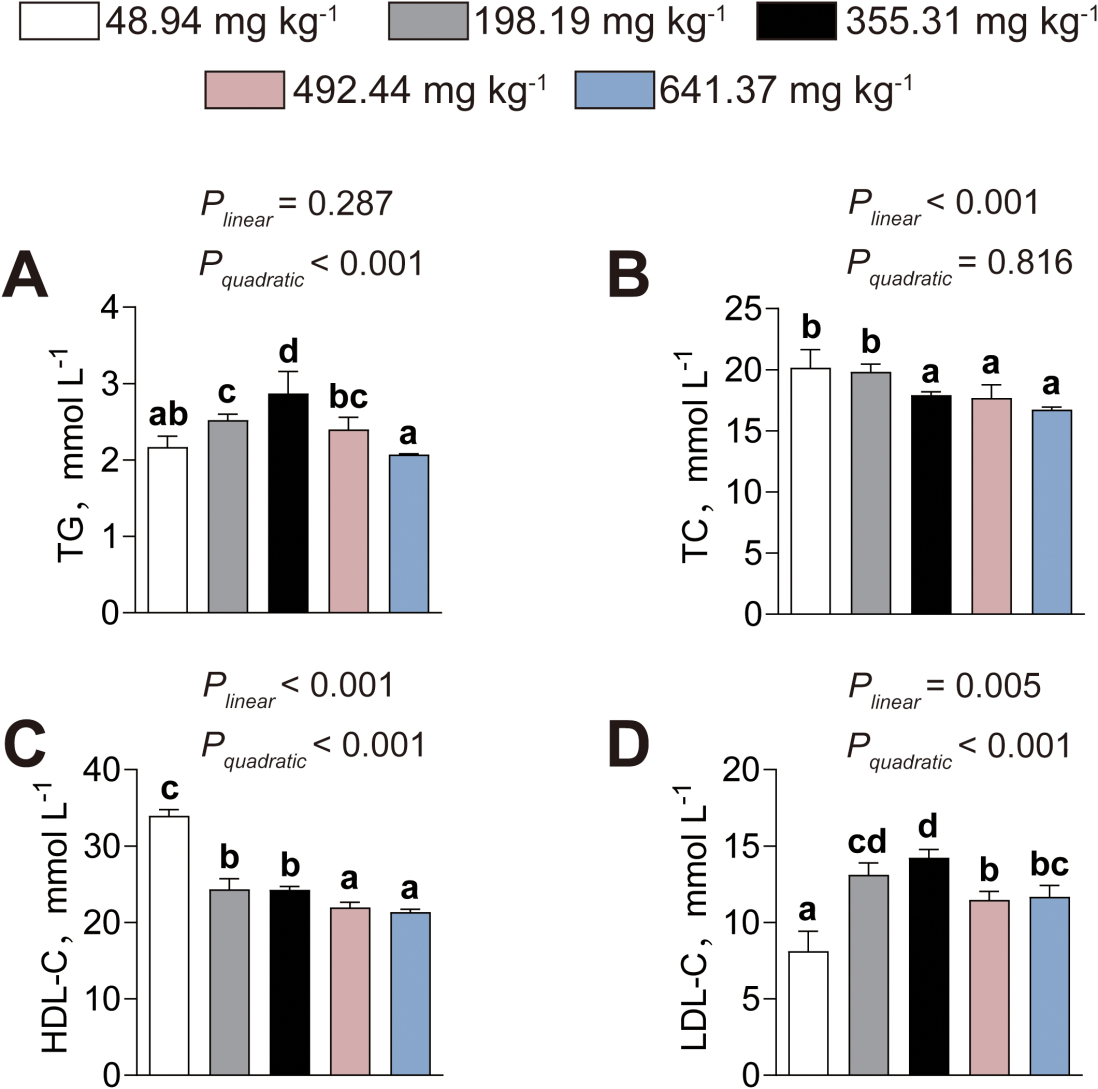
Effects of dietary histamine levels on the serum lipid parameters of juvenile American eels. **(A)** Serum TG contents. **(B)** Serum TC contents. **(C)** Serum HDL-C concentration. **(D)** Serum LDL-C concentration. Values are shown as mean ± S.D. (n = 3). Letters (a–d) denote significance at *P* < 0.05.

Dietary histamine–induced alterations in serum lipid metabolism are shown in **Figure 3**. Serum TG content showed quadratic trend, characterized by an initial increase followed by a subsequent decline as dietary histamine levels rose (*P_linear_*= 0.287, *P_quadratic_*< 0.001) (**Figure 3A**). American eels fed the diet containing 355.31 mg kg^-1^ histamine exhibited the highest serum TG content among these fed diets containing different histamine levels (**Figure 3A**). Serum TC level exhibited significant linear decreases with increasing dietary histamine, with significant reductions observed beginning at 355.31 mg kg^-1^ (*P_linear_*< 0.001, *P_quadratic_*= 0.816) (**Figure 3B**). With increasing dietary histamine, serum HDL-C level decreased both linearly and quadratically (*P_linear_*< 0.001, *P_quadratic_*< 0.001), whereas LDL-C level exhibited a non-linear quadratic trend (*P_linear_*= 0.005, *P_quadratic_*< 0.001), initially rising before declining at higher concentrations (**Figures 3C, D**). Thus, excessive dietary histamine levels induce abnormal serum lipid metabolism in American eels.

### 3.4 Intestinal morphology

**Figure 4:**
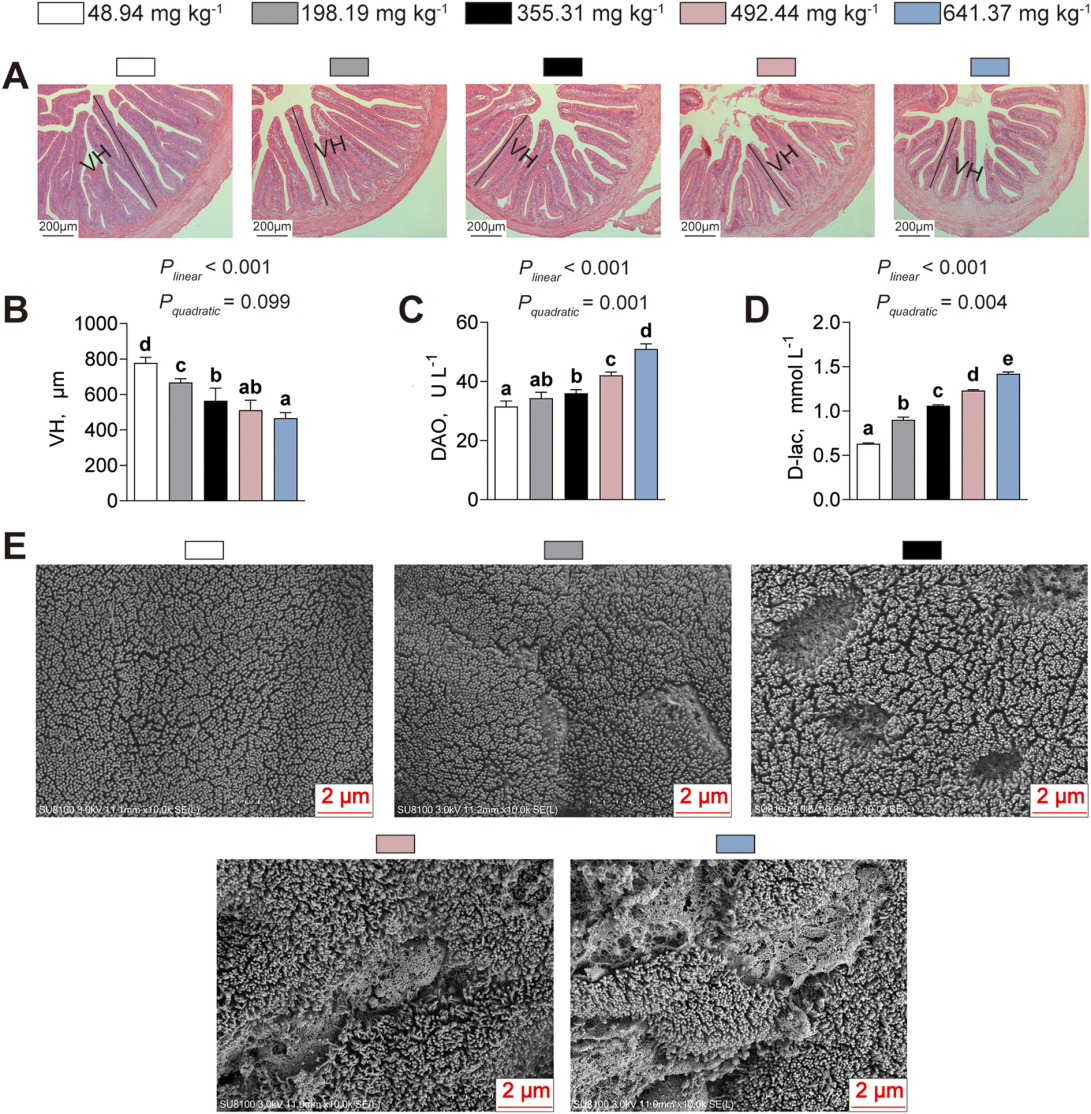
Effects of dietary histamine levels on the intestinal structure of juvenile American eels. **(A)** Representative microphotograph of hematoxylin & eosin (H&E) staining. Scale bar, 200 μm. VH, villus height. **(B)** Statistical analysis of villi height, related to **Figure 4A**. **(C)** Serum DAO activity. **(D)** Serum D-lac concentration. **(E)** Representative ultrastructural observation of the intestinal microvilli, scale bar = 2 μm. Values are shown as mean ± S.D. (n = 3). Letters (a–e) denote significance at *P* < 0.05.

Subsequently, the effect of dietary histamine on intestinal morphology was assessed (**Figure 4**). Villus height (VH) decreased linearly with increasing dietary histamine (*P_linear_*< 0.001, *P_quadratic_*= 0.099), with significant reductions initiated at 198.19 mg kg^-1^ (**Figures 4A, B**). Serum DAO activity (*P_linear_*< 0.001, *P_quadratic_*= 0.001) and D–lac level (*P_linear_*< 0.001, *P_quadratic_*= 0.004) increased both linearly and quadratically as dietary histamine levels increased (**Figures 4C, D**). Moreover, SEM analysis indicated that American eels received diets with histamine exceeding 355.31 mg kg^-1^ possessed intestinal microvilli arranged in a markedly sparser configuration compared to those fed the control diet (**Figure 4E**). Thus, excessive dietary histamine levels compromise intestinal mucosal integrity of American eels.

### 3.5 Intestinal antioxidant capacity

**Figure 5:**
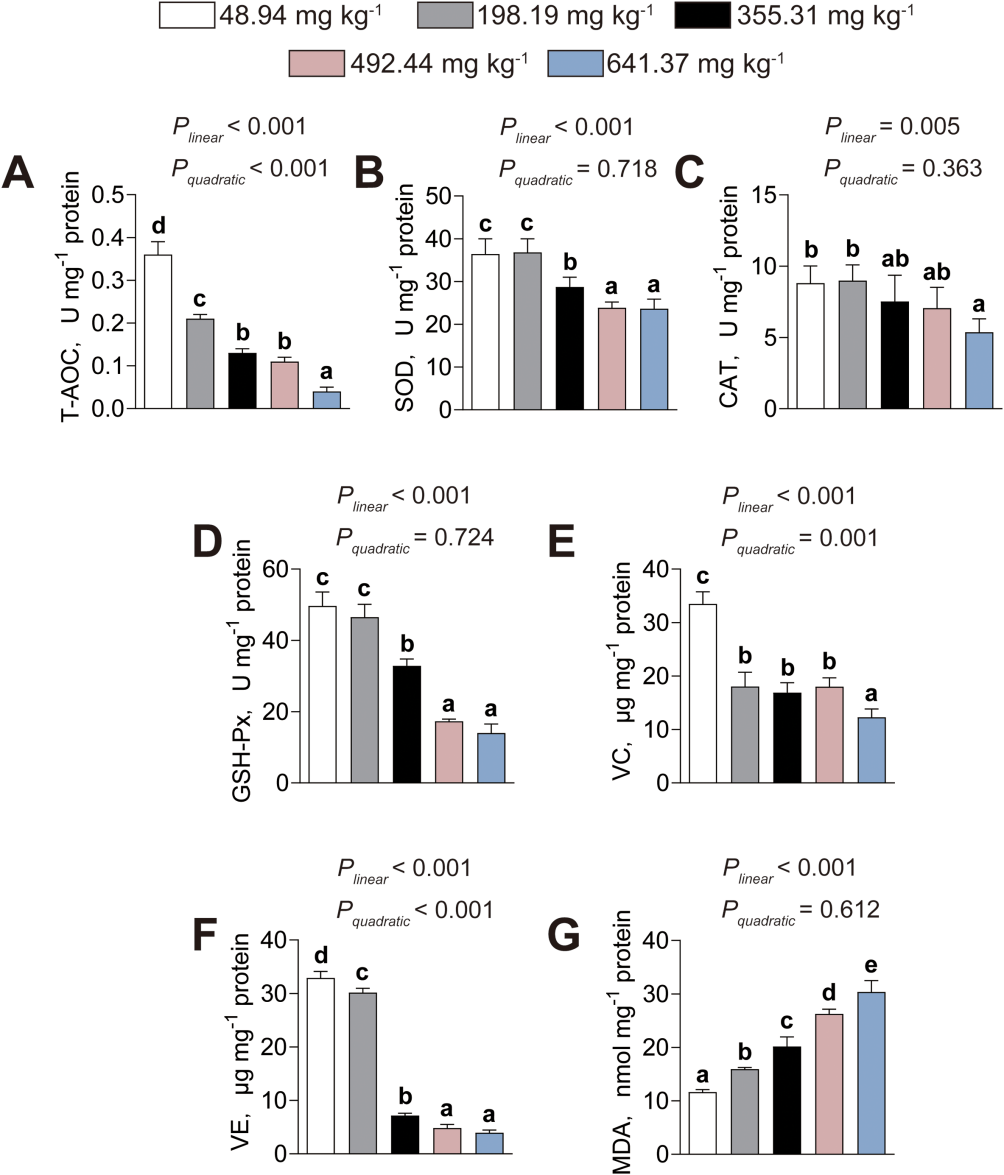
Effects of dietary histamine levels on the intestinal antioxidant capacity of juvenile American eels. **(A)** T-AOC. **(B)** SOD activity. **(C)** CAT activity. **(D)** GSH-Px activity. **(E)** Vitamin C (VC) level. **(F)** Vitamin E (VE) level. **(G)** MDA level. Values are shown as mean ± S.D. (n = 3). Letters (a–e) denote significance at *P* < 0.05.

The influence of dietary histamine levels on the intestinal antioxidant capacity of American eels is presented in **Figure 5**. The intestinal T–AOC activity (*P_linear_*< 0.001, *P_quadratic_*< 0.001), along with the levels of intestinal vitamin C (VC, *P_linear_*< 0.001, *P_quadratic_*= 0.001) and vitamin E (VE, *P_linear_*< 0.001, *P_quadratic_*< 0.001), decreased both linearly and quadratically as dietary histamine levels increased (**Figure 5**). The activities of intestinal SOD (*P_linear_*< 0.001, *P_quadratic_*= 0.718), CAT (*P_linear_*< 0.001, *P_quadratic_*= 0.363), and GSH-Px (*P_linear_*< 0.001, *P_quadratic_*= 0.724) decreased linearly as dietary histamine levels increased, with significant reductions initiated at 355.31 mg kg^-1^, 641.37 mg kg^-1^, 355.31 mg kg^-1^, respectively (**Figures 5B–D**). In contrast, intestinal MDA content increased linearly as dietary histamine levels increased (*P_linear_*< 0.001, *P_quadratic_*= 0.612), with a significant elevation initiated at 198.19 mg kg^-1^ (**Figure 5G**). Therefore, excessive dietary histamine inhibits antioxidant defenses and induces lipid peroxidation in the intestine.

### 3.6 Intestinal digestive enzymes activities

**Figure 6:**
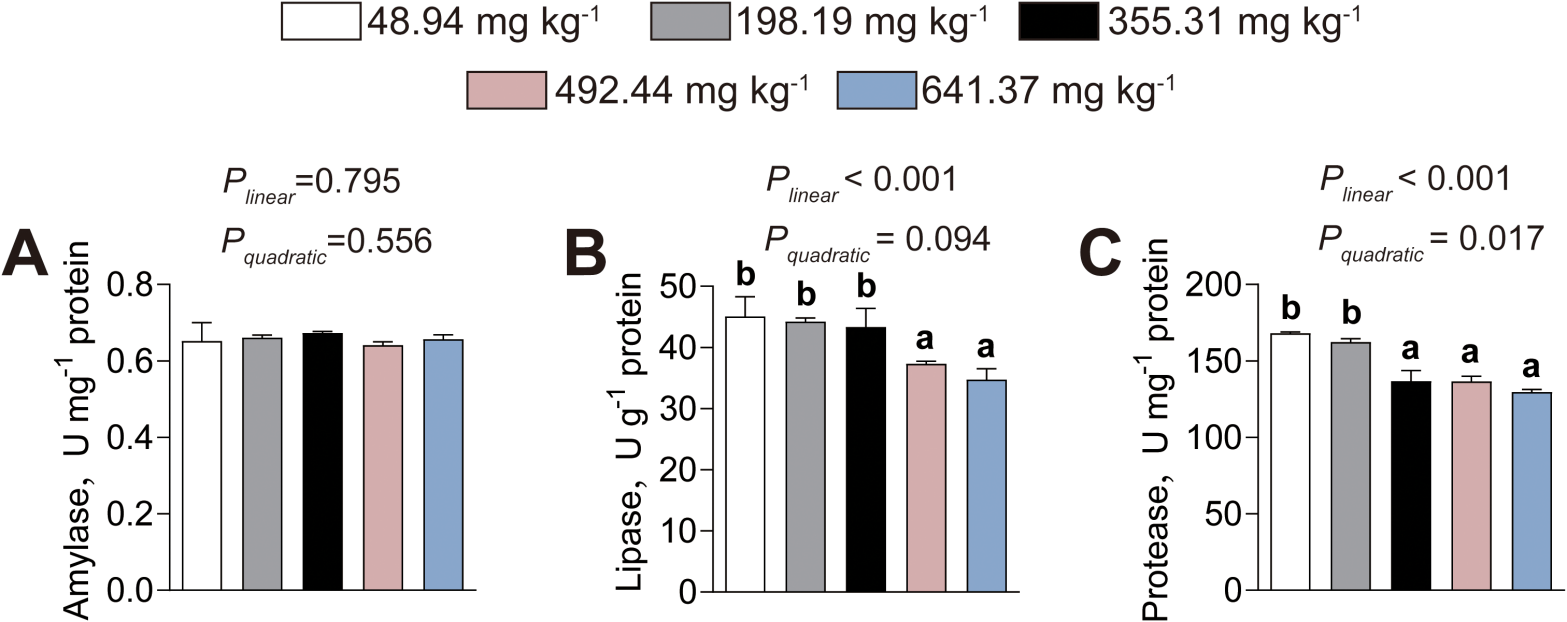
Effects of dietary histamine levels on the activities of intestinal digestive enzymes in juvenile American eels. **(A)** Amylase activity. **(B)** Lipase activity. **(C)** Protease activity. Values are shown as mean ± S.D. (n = 3). Letters (a–e) denote significance at *P* < 0.05.

The effects of dietary histamine levels on intestinal digestive enzyme activities are shown in **Figure 6**. Dietary histamine levels did not significantly affect intestinal amylase activity (**Figure 6A**). The lipase activity (*P_linear_*< 0.001, *P_quadratic_*= 0.094) decreased linearly, whereas protease activity (*P_linear_*< 0.001, *P_quadratic_*= 0.017) decreased both linearly and quadratically, as dietary histamine levels increased, with significant reductions initiated at 492.44 mg kg^-1^ and 355.31 mg kg^-1^, respectively (**Figures 6B, C**). Consequently, excessive dietary histamine inhibits intestinal digestive enzyme activities of American eels.

### 3.7 Intestinal microbial composition

**Figure 7:**
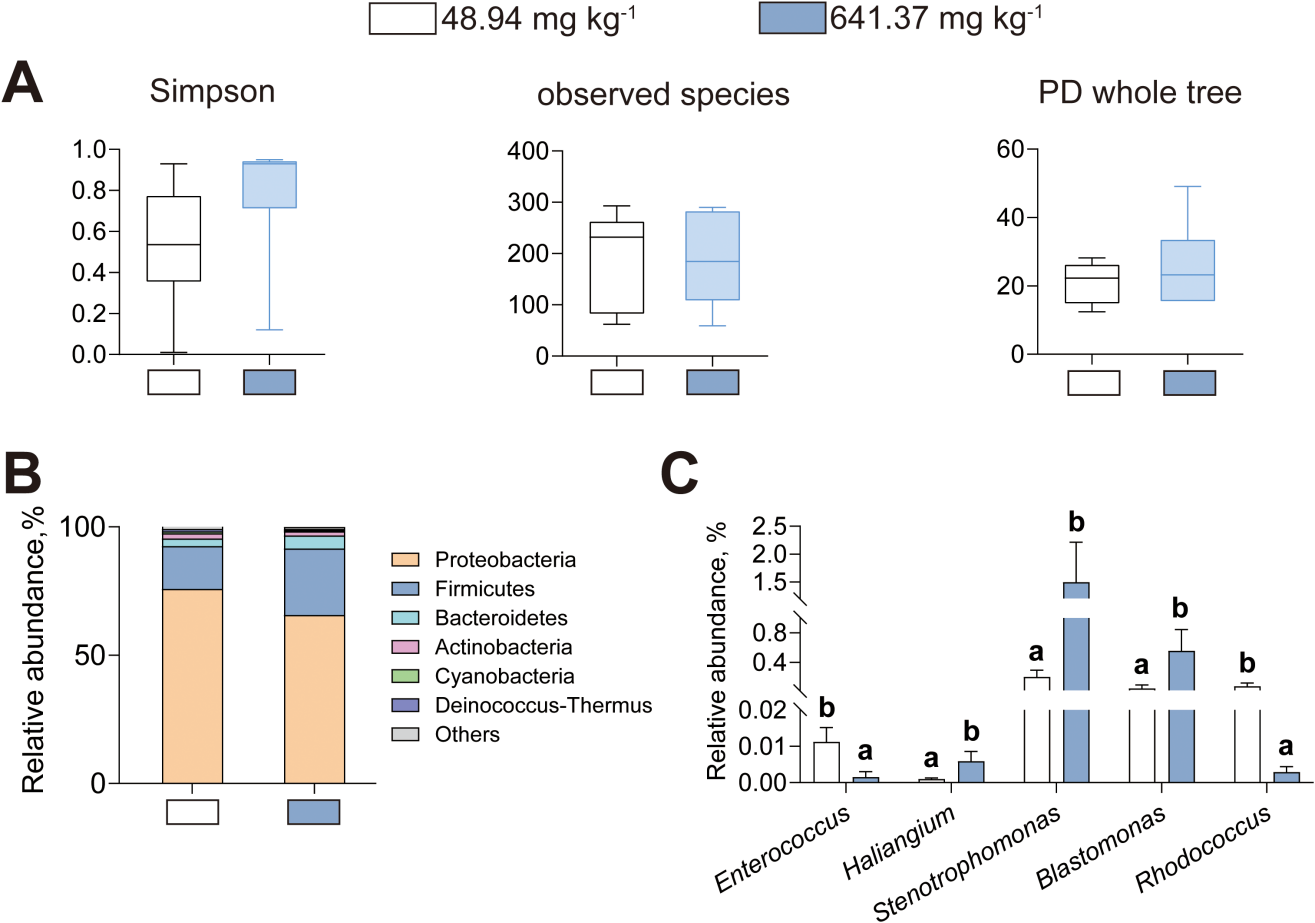
Effects of dietary histamine levels on the intestinal microbial compositions. **(A)** The intestinal flora alpha diversity. **(B)** The relative abundance of the intestinal bacterial phyla. **(C)** The analysis of the intestinal bacterial genus. Letters (a, b) denote significance at *P* < 0.05.

Given the essential role of the intestinal microorganism in intestinal health, microbial profiles were compared between American eels received diets containing either 48.94 or 641.37 mg kg^-1^ histamine (**Figure 7**). Alpha diversity indices, including Simpson index, observed species richness, and PD whole tree, did not differ between American eels received those two diets (**Figure 7A**). The predominant intestinal microbial phyla in American eels were Proteobacteria, Firmicutes, Bacteroidetes, Actinobacteria, and Cyanobacteria in American eels, the relative abundance of these phyla exhibited no significant difference between American eels fed those two diets (**Figure 7B**). Compared to the control diet, a dietary histamine level of 641.37 mg kg^-1^ decreased abundance of *Enterococcus* and *Rhodococcus*, along with increased levels of *Haliangium*, *Stenotrophomonas*, and *Blastomonas* (**Figure 7C**). Therefore, excessive dietary histamine disrupts the balance of intestinal microbial composition in American eels.

### 3.8 Liver histology

**Figure 8:**
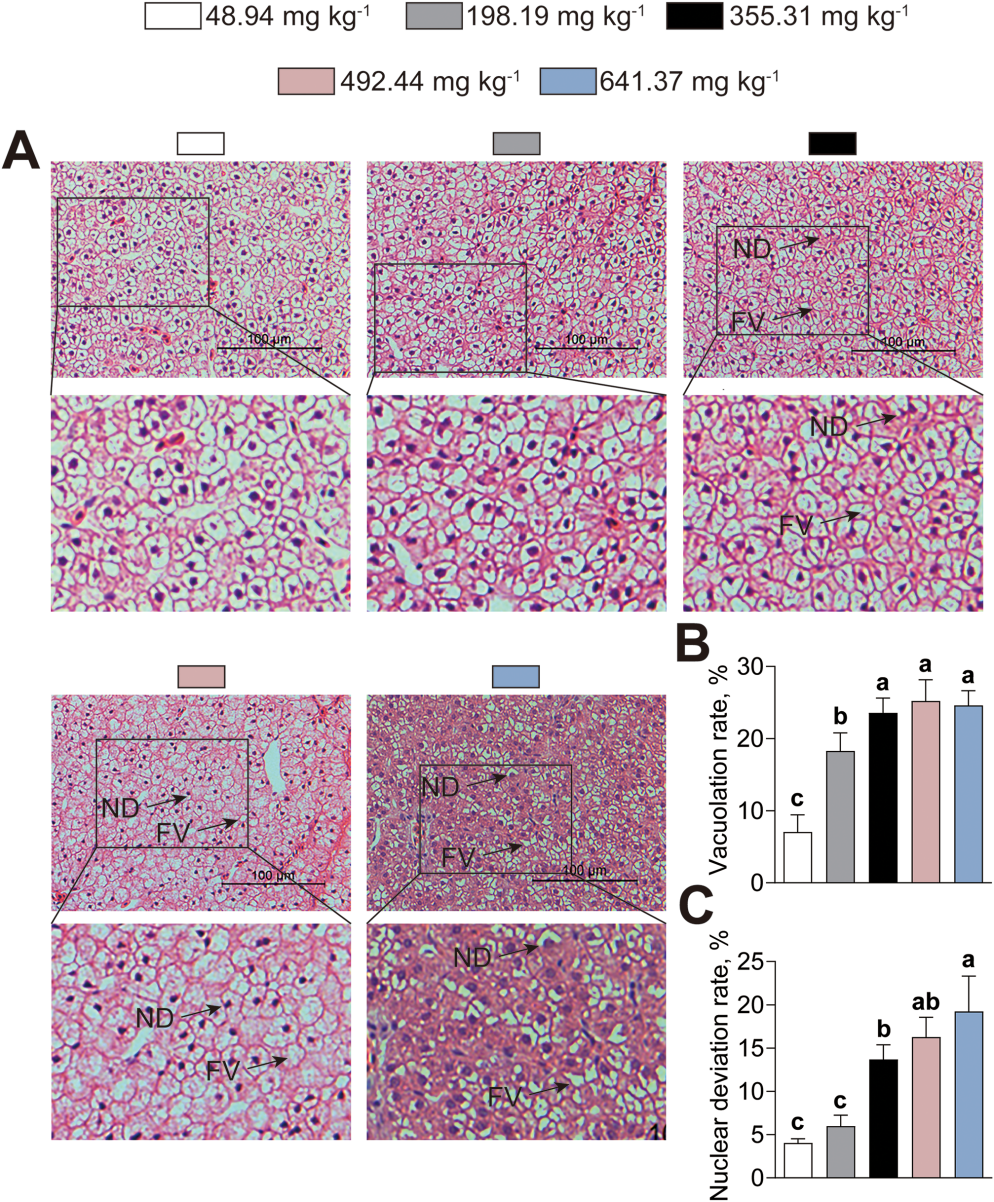
Effects of dietary histamine levels on the hepatic structure of juvenile American eels. **(A)** Representative microphotograph of hematoxylin & eosin (H&E) staining. Scale bar, 100 μm. ND, nuclear displacement; FV, fat vacuoles. **(B)** Statistical analysis of vacuolation rate, related to **Figure 8A**. **(C)** Statistical analysis of nuclear deviation rate, related to **Figure 8A**. Values are shown as mean ± S.D. (n = 3). Letters (a–e) denote significance at *P* < 0.05.

The alterations in hepatic structural integrity induced by dietary histamine were evaluated through histology (**Figure 8**). Hepatocytes from American eels fed diets with either 48.94 or 198.19 mg kg^-1^ histamine displayed distinct boundaries with large, centrally located spherical nuclei clearly visible (**Figure 8**). In contrast, American eels fed diets with histamine levels exceeding 355.31 mg kg^-1^ exhibited a higher prevalence of hepatocytes presenting nuclear displacement and vacuolar degeneration (**Figure 8**). Additionally, American eels fed the diet containing 641.37 mg kg^-1^ histamine showed incomplete lobular architecture along with indistinct cellular boundaries (**Figure 8**). Thus, excessive dietary histamine levels induce hepatic injure of American eels.

### 3.9 Serum GOT and GPT activities

**Figure 9:**
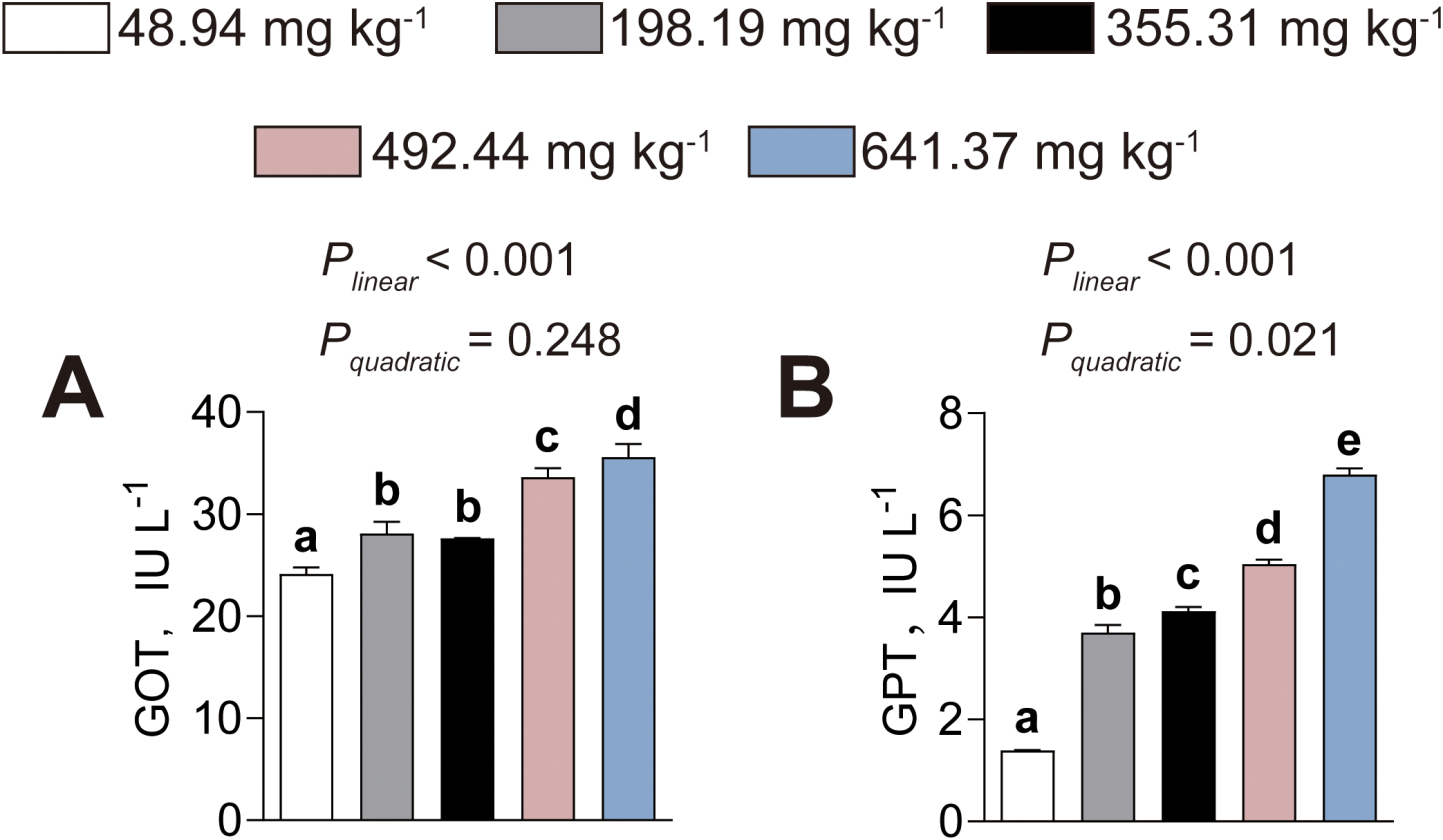
Effects of dietary histamine levels on the activities of serum GOT **(A)** and GPT **(B)** of juvenile American eels. Values are shown as mean ± S.D. (n = 3). Letters (a–e) denote significance at *P* < 0.05.

Serum GOT activity (*P_linear_*< 0.001, *P_quadratic_*= 0.248) elevated linearly, whereas serum GPT activity (*P_linear_*< 0.001, *P_quadratic_*= 0.021) elevated both linearly and quadratically, as dietary histamine levels increased, with significant elevations initiated at 198.19 mg kg^-1^ (**Figures 9A, B**). Thus, these findings further confirmed that excessive dietary histamine levels destroyed the liver of American eels.

### 3.10 Hepatic antioxidant capacity

**Figure 10:**
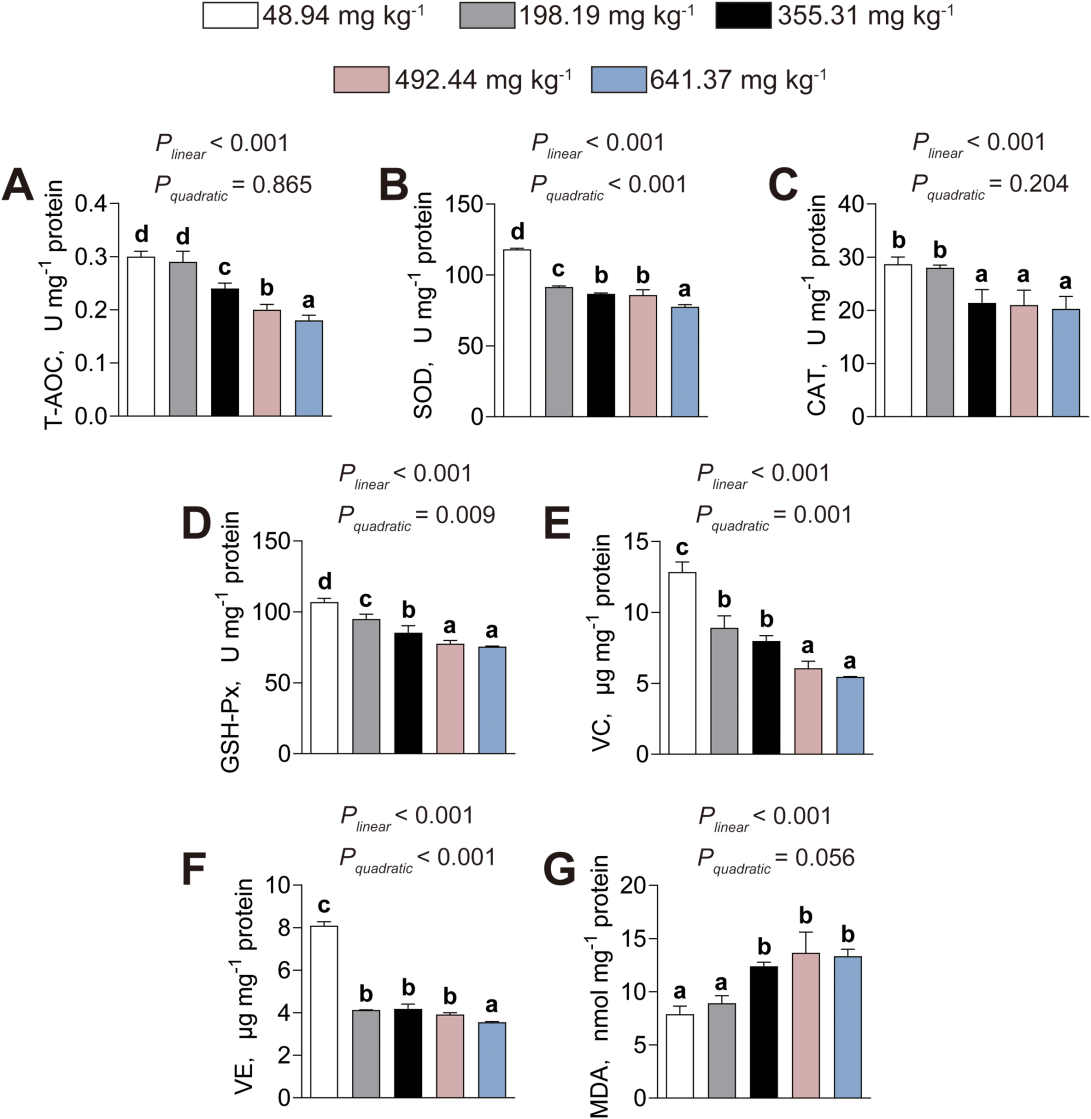
Effects of dietary histamine levels on the hepatic antioxidant capacity of juvenile American eels. **(A)** T-AOC. **(B)** SOD activity. **(C)** CAT activity. **(D)** GSH-Px activity. **(E)** Vitamin C (VC) level. **(F)** Vitamin E (VE) level. **(G)** MDA level. Values are shown as mean ± S.D. (n = 3). Letters (a–e) denote significance at *P* < 0.05.

The influence of dietary histamine levels on hepatic antioxidant capacity of American eels is illustrated in **Figure 10**. The activities of hepatic T–AOC (*P_linear_*< 0.001, *P_quadratic_*= 0.865) and CAT (*P_linear_*< 0.001, *P_quadratic_*= 0.204) decreased linearly as dietary histamine levels increased, with significant reductions initiated at 355.31 mg kg^-1^ (**Figures 10A, C**). The activities of hepatic SOD (*P_linear_*< 0.001, *P_quadratic_*< 0.001) and GSH-Px (*P_linear_*< 0.001, *P_quadratic_*= 0.009), along with the levels of hepatic VC (*P_linear_*< 0.001, *P_quadratic_*= 0.001) and VE (*P_linear_*< 0.001, *P_quadratic_*< 0.001), decreased both linearly and quadratically as dietary histamine levels increased, with significant reductions initiated at 198.19 mg kg^-1^ (**Figure 10**). In contrast, hepatic MDA content increased linearly as dietary histamine levels increased (*P_linear_*< 0.001, *P_quadratic_*= 0.056), with a significant elevation initiated at 355.31 mg kg^-1^ (**Figure 10G**). These findings indicate that excessive dietary histamine compromises hepatic antioxidant defenses and promotes lipid peroxidation in the liver of American eels.

### 3.11 Metabolomics analysis of the liver

**Figure 11:**
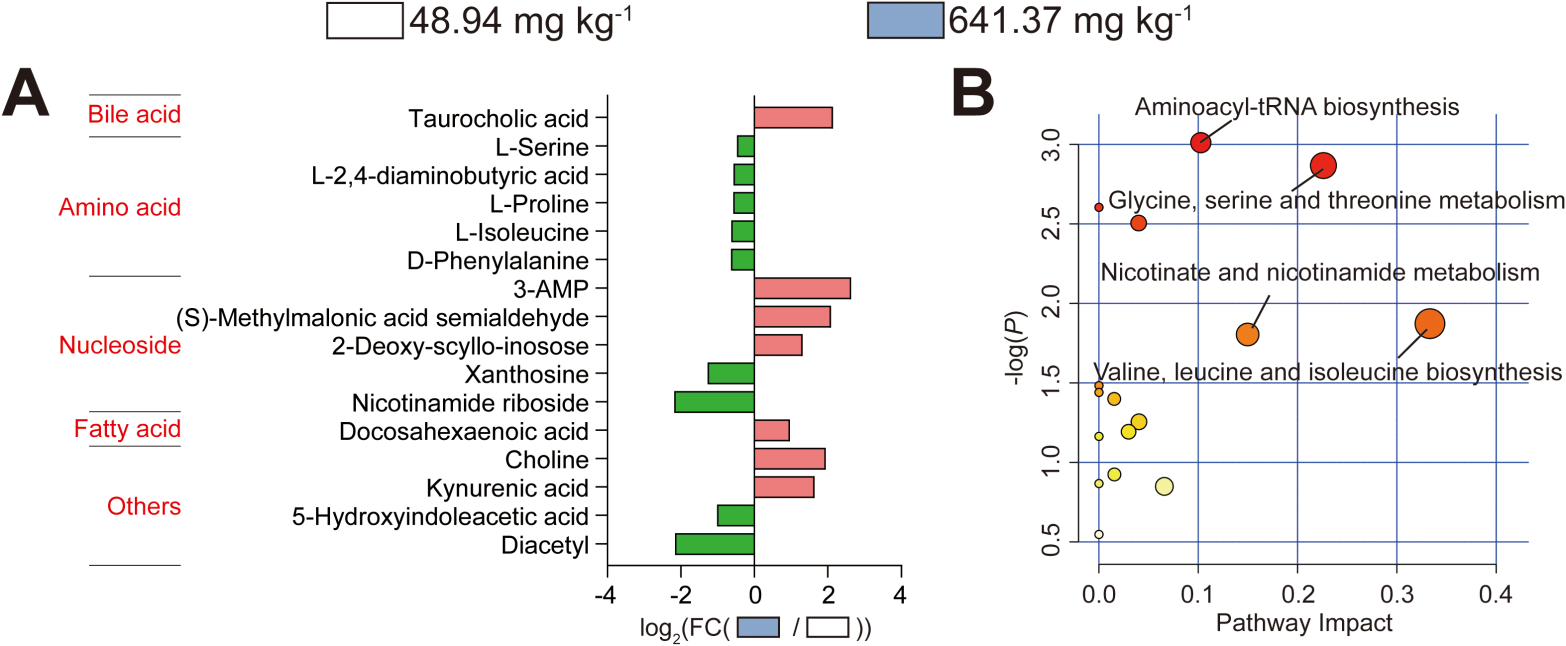
Metabolomics analysis of the liver in juvenile American eels fed with different levels of histamine. **(A)** Differential metabolites (DMs) visualized as a histogram. **(B)** KEGG enrichment of DMs.

Finally, we performed hepatic metabolomics analysis comparing American eels fed diets containing either 48.94 or 641.37 mg kg^-1^ histamine to uncover the underlying metabolic alterations in the liver induced by excessive dietary histamine levels (**Figure 11**). Compared to American eels fed the control diet, a total of 16 differential metabolites (DMs) were detected in those received the diet with 641.37 mg kg^-1^ histamine (**Figure 11A**). Among these DMs, levels of 7 metabolites were elevated and levels of 9 metabolites were reduced in American eels fed the diet containing 641.37 mg kg^-1^ histamine compared to those receiving the control diet (**Figure 11A**). Furthermore, KEGG pathway enrichment analysis of these DMs highlighted significant perturbations in aminoacyl–tRNA biosynthesis, glycine, serine, and threonine metabolism, nicotinate and nicotinamide metabolism, valine, leucine, and isoleucine biosynthesis were significantly affected (**Figure 11B**).

## 4 Discussion

This study firstly explored the influences of different dietary histamine levels on growth performance and feed utilization of American eels. Growth performance was evaluated using FBW, WGR, and SGR, while FE was used to reflect feed utilization (Xu et al., 2025; Yin et al., 2024). This study showed that excessive dietary histamine leaded to significant reductions in FBW, WGR, SGR, and FE, which indicated that excessive dietary histamine inhibited growth performance and feed utilization. Likewise, our previous study demonstrated that excessive dietary histamine suppressed growth performance and feed utilization of American eels (Zhai et al., 2020). Similar negative responses have also been observed in other fish species, including grouper, striped catfish, and yellow catfish (Liu et al., 2022, Liu et al., 2021b; He et al., 2018). However, Zhang et al. (2023) reported that hybrid grouper (*Epinephelus fuscoguttatus*♀×*Epinephelus lanceolatus*♂) showed no significant changes in growth performance or feed utilization under high histamine feeding. Opstvedt et al. (2000) also demonstrated that dietary histamine did not affect growth of Atlantic salmon (*Salmo salar* L.). These inconsistent findings imply that sensitivity to dietary histamine is highly species–specific, potentially due to interspecific differences in the capacity of the intestine and liver to metabolize histamine (Schnedl and Enko, 2021). Furthermore, regression analysis of WGR estimated a maximum tolerable dietary histamine level of 260.11 mg kg^-1^ for American eels, a value comparable to that reported for striped catfish (Liu et al., 2022). Nevertheless, previous studies indicated that grouper tolerate significantly higher dietary histamine levels, reaching up to 2500 mg kg^-1^, which contrasts markedly with the findings for American eels (Liu et al., 2021b). Species with high dietary histamine tolerance, such as grouper, may possess more efficient enzymatic systems for histamine degradation (Jochum, 2024). Conversely, American eels and striped catfish appear to have limited histamine detoxification capacity, making them more susceptible to histamine toxicity at lower dietary levels (Jochum, 2024). Therefore, these findings underscore the importance of establishing species–specific dietary histamine limits rather than applying universal standards across aquaculture species.

Immune function was assessed using serum AKP and ACP activities, as well as serum IgM and C3 concentrations (Xu et al., 2026). Both AKP and ACP serve as indicators of non–specific immunity, IgM is central to humoral immune responses, and C3 contributes to bactericidal activity and complement activation (Etayo et al., 2024; Gao et al., 2024; Wu et al., 2022). This study uncovered that excessive dietary histamine inhibited the activities of serum AKP and decreased the levels of serum C3 and IgM, which indicated that excessive dietary histamine suppressed immune function in American eels. Similarly, Liu et al. (2021b) demonstrated that excessive dietary histamine decreased serum AKP and ACP activities in grouper. Additionally, Cheng et al. (2026) reported that high dietary histamine levels inhibited serum AKP activities and reduced serum C3 concentrations, suggesting that immune suppression represents a conserved response to excessive histamine intake. Histamine is a bioactive amine capable of modulating immune cell activity through histamine receptor–mediated signaling and regulation of inflammatory pathways, including NF–κB signaling (Cheng et al., 2026; Kusiak and Brady, 2022). Excessive histamine exposure may therefore disturb immune homeostasis by altering transcriptional regulation of immune-related genes, ultimately leading to reduced synthesis of complement components and immunoglobulins (Yu et al., 2020). Importantly, immune dysfunction may increase susceptibility to oxidative stress and tissue injury, which are further discussed below. Thus, excessive dietary histamine appears to compromise systemic defense capacity in American eels.

The intestine is a vital organ in fish, functioning as the principal site for nutrient digestion and absorption. The liver functions as the metabolic center and is essential for maintaining energy homeostasis and physiological balance (Xu et al., 2025). Structural integrity and antioxidant capacity are key indicators of intestinal and hepatic health. In the present study, excessive dietary histamine induced marked alterations in both intestinal and hepatic morphology, accompanied by profound disturbances in antioxidant status, suggesting a coordinated pathophysiological response rather than isolated tissue damage. Histological analysis revealed high dietary histamine shortened villi and disrupted microvilli in the intestine, indicating a reduced absorptive surface area and compromised barrier integrity (Yin et al., 2024). These structural impairments were further supported by elevated serum DAO activity and D–lac levels, established biomarkers of increased intestinal permeability (Yin et al., 2024). Together, these findings suggest that excessive dietary histamine disrupts epithelial integrity and leads to enhanced mucosal permeability in American eels. Similar negative influences of dietary histamine on intestinal morphology have been observed in striped catfish, grouper, and yellow catfish (Li et al., 2021; Liu et al., 2021b, Liu et al., 2022). The intestine–liver axis provides a mechanistic link between intestinal barrier dysfunction and hepatic injury (Pabst et al., 2023). Increased intestinal permeability may facilitate the translocation of histamine and other metabolites into systemic circulation, thereby imposing metabolic and inflammatory stress on the liver. Consistent with this hypothesis, it was observed that excessive dietary histamine induced nuclear displacement, vacuolar degeneration, and indistinct hepatocellular boundaries. Moreover, excessive dietary histamine elevated serum GOT and GPT activities, suggesting high dietary histamine levels cause hepatocellular damage and compromised membrane integrity (McGill, 2016). Similarly, Li et al. (2018) reported that excessive dietary histamine damage to the hepatic structure of yellow catfish. Given that histamine acts as a potent bioactive amine and a primary mediator of inflammation, these findings suggest that hepatic structural damage represents more than a localized toxic response. Instead, it is likely a systemic consequence secondary to dietary histamine-induced intestinal barrier failure.

Furthermore, our research revealed that American eels fed high–histamine diets exhibited attenuated antioxidant enzymatic activities, decreased VC and VE levels, and increased MDA content in both the intestine and liver. T–AOC provides an integrated measure of antioxidant status (Xu et al., 2025). SOD catalyzes the dismutation of superoxide radicals to hydrogen peroxide, which is subsequently detoxified by CAT and GSH–Px, thereby preventing oxidative damage (Gulcin, 2020). The coordinated antioxidant activity can restrain lipid peroxidation, as reflected by lower MDA levels (Xu et al., 2026). VC acts as a water–soluble radical scavenger, while VE functions as an effective lipid–soluble antioxidant that neutralize reactive oxygen species and lipid peroxides (Rychter et al., 2022). Collectively, these results demonstrated that excessive dietary histamine impairs antioxidant defenses and promotes lipid peroxidation in the intestine and liver of American eels. Consistent with these findings, striped catfish fed high–histamine diets exhibited decreased antioxidant capacity and elevated lipid peroxidation in the intestine, and excessive dietary histamine also contributed to hepatic lipid peroxidation in grouper (Liu et al., 2022, Liu et al., 2021b). The observed reduction in antioxidant capacity is likely attributed to the excessive accumulation of histamine, which may trigger reactive oxygen species (ROS) overproduction by activating inflammatory signaling (Branco et al., 2018). To maintain redox homeostasis, the organism rapidly consumes enzymatic and non–enzymatic antioxidant defenses, leading to their depletion. Consequently, unneutralized ROS compromise membrane integrity by oxidizing polyunsaturated fatty acids (Bodega et al., 2019). This oxidative degradation severely compromises the fluidity and integrity of cellular membranes, which may serves as a primary driver of the intestinal and hepatic structural damage observed in this study (Bodega et al., 2019). Therefore, oxidative stress likely serves as a central mediator linking excessive dietary histamine to both structural deterioration and functional impairment in these organs. Collectively, the present findings support a mechanistic framework in which excessive dietary histamine disrupts intestinal barrier integrity, enhances systemic metabolic and oxidative stress, and ultimately impairs hepatic structure and antioxidant defenses. This integrative response highlights oxidative imbalance and barrier dysfunction as key drivers of dietary histamine-induced pathophysiology in American eels.

The intestinal microorganism is integral to nutrient metabolism, immune modulation, and barrier maintenance. Under normal physiological conditions, the intestinal flora maintains a stable symbiotic relationship with the host and remains relatively stable in composition and abundance. However, external stressors can disrupt microbial homeostasis, leading to metabolic dysregulation and intestinal disorders such as inflammation (Xu et al., 2026). In this research, the predominant intestinal microbial phyla in American eels were Proteobacteria and Firmicutes, in accord with previous reports for this species (Xu et al., 2024). Comparatively, the predominant phyla in European eels (*Anguilla anguilla*) include Spirochaetae, Fusobacteria, and Proteobacteria while marbled eels (*Anguilla marmorata*) are primarily dominated by Fusobacteria, Firmicutes, and Tenericutes (Lin et al., 2019; Shi et al., 2020). These observations highlight significant interspecific variations in the dominant gut bacterial communities of eels. In this study, dietary histamine at 641.37 mg/kg significantly decreased the abundances of *Enterococcus* and *Rhodococcus*, while elevating the levels of *Haliangium*, *Stenotrophomonas*, and *Blastomonas*. *Enterococcus* and *Rhodococcus* are generally considered beneficial intestinal bacteria in fish (Kokou et al., 2019; Matsuura et al., 2017). *Enterococcus* is known to protect the intestinal barrier and enhances the intestinal resistance to pathogenic infections (Matsuura et al., 2017). *Rhodococcus* has been detected in the intestines of snakehead (*Ophiocephalus argus Cantor*) and European sea bass (*Dicentrarchus labrax*), which could promote digestion and absorption and help resist pathogen invasion (Kokou et al., 2019). Conversely, *Stenotrophomonas* is an opportunistic pathogen capable of inducing intestinal inflammation and barrier dysfunction (He et al., 2023). Currently evidence regarding the roles of *Haliangium* and *Blastomonas* in intestinal homeostasis and fish health remains limited. Given their environmental origins, they are likely transient passengers rather than permanent colonizers of the gut ecosystem (McIlroy et al., 2016; Webster et al., 2021). Overall, excessive histamine shifted the intestinal flora of American eels toward a higher relative abundance of potential pathogens and a lower relative abundance of beneficial bacteria.

To further elucidate the mechanisms underlying hepatic impairment, untargeted metabolomic analysis was performed. Classification of differential metabolites and pathway enrichment analysis indicated that excessive dietary histamine primarily affected hepatic amino acid and nucleotide metabolism in juvenile American eels. Notably, reductions in L–isoleucine and L–serine indicate suppression of aminoacyl–tRNA biosynthesis, a pathway essential for translational efficiency and protein synthesis, under the treatment of histamine (Tennakoon and Cui, 2024). Aminoacyl–tRNAs, formed via the esterification of specific amino acids catalyzed by aminoacyl–tRNA synthetases, are essential for protein biosynthesis (Luo et al., 2025). Impaired aminoacyl–tRNA formation may compromise hepatic protein turnover and enzymatic synthesis, thereby weakening metabolic and antioxidant defenses. Furthermore, since serine can be converted into glycine by serine hydroxymethyltransferase, the observed reduction of L–serine also implies an inhibition of the glycine, serine, and threonine metabolism pathway (Zhou et al., 2024). This pathway is essential for inhibiting oxidative stress, enhancing immunity, and regulating lipid metabolism (Zhou et al., 2024). Disruption of this pathway may therefore exacerbate oxidative stress, consistent with the observed decline in antioxidant capacity. Methylmalonic acid semialdehyde and L–isoleucine participate in valine, leucine, and isoleucine biosynthesis (Tan et al., 2025). In the present study, excessive dietary histamine decreased L–isoleucine and increased methylmalonic acid semialdehyde, indicating weakened valine, leucine, and isoleucine biosynthesis. Given the established roles of valine, leucine, and isoleucine in regulating immune function, glucose oxidation, and lipid metabolism, such disturbances may contribute to both the compromised immune responses and dysregulated lipid profiles observed in this study (Yuan et al., 2020). Furthermore, at a dietary histamine concentration of 641.37 mg/kg, the downregulation of nicotinamide riboside suggested a suppression of nicotinate and nicotinamide metabolism (Hofer et al., 2021). This pathway is involved in inflammatory responses, and nicotinamide riboside has been reported to alleviate hepatic histopathological injury and dysfunction while reducing lipid accumulation (Guo et al., 2022). Collectively, these metabolomic alterations suggest that excessive dietary histamine induces a shift toward impaired amino acid utilization, compromised protein synthesis, disrupted redox regulation, and altered energy metabolism. Rather than representing secondary consequences, these metabolic disturbances likely constitute a central driver of hepatic oxidative imbalance, lipid dysregulation, and structural injury. Thus, the hepatic metabolic signature identified here provides mechanistic support for the biochemical and histopathological changes observed in American eels exposed to excessive dietary histamine.

## 5 Conclusion

In conclusion, this study delineates the tolerance limit and systemic toxicity of dietary histamine in American eels. Excessive dietary histamine induced growth retardation, suppressed immune function, decreased intestinal digestive enzyme activities, and compromised intestinal and hepatic mucosal integrity and antioxidant defenses in American eels. Omics analyses further showed that high dietary histamine reshaped the intestinal microbiota by reducing probiotic abundance but promoting pathogens colonization, while concurrently altering hepatic amino acid and nucleotide metabolism. Broken–line regression analysis of WGR determined a maximum tolerable dietary histamine threshold of 260.11 mg kg^-1^. Overall, this work offers a comprehensive assessment for understanding dietary histamine toxicity in American eels, highlights the need to restrict histamine levels in feeds, and provides a scientific basis for optimizing nutritional strategies in sustainable aquaculture. While this study elucidates the pathophysiological consequences of dietary histamine exposure in American eels, future investigations are warranted to determine whether these physiological disturbances translate into compromised flesh quality.

## Data Availability

The datasets presented in this study are deposited in the NCBI, accession number PRJNA1415989.
